# 3,3′-Difluoro-4,4′-(*p*-phenyl­enedi­oxy)dibenzonitrile

**DOI:** 10.1107/S1600536809035247

**Published:** 2009-09-05

**Authors:** Jixu Zhang, Jiayi Wu, Jianfeng Wang, Yiming Li, Shuping Luo

**Affiliations:** a State Key Laboratory Breeding Base of Green Chemistry-Synthesis Technology, Zhejiang University of Technology, Hangzhou 310014, People’s Republic of China

## Abstract

The title compound, C_20_H_10_F_2_N_2_O_2_, was synthesized from hydro­quinone and 3,4-difluoro­benzonitrile. The centroid of the central aromatic ring is on a crystallographic center of inversion. The dihedral angle between the central and terminal rings is 77.8 (3)°. In the crystal, chains linked by C—H⋯N bond occur.

## Related literature

For the herbicidal actvity of hydro­quinone derivatives, see: Bao *et al.* (2007 ▶). For related structures, see: Sørensen & Stuhr-Hansen (2009 ▶); Luo *et al.* (2009 ▶); Liu (2002 ▶).
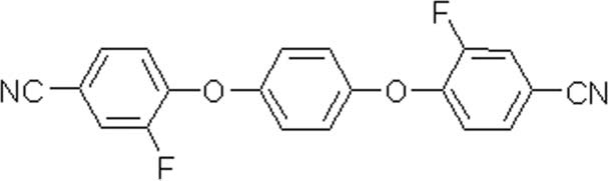


## Experimental

### 

#### Crystal data

C_20_H_10_F_2_N_2_O_2_
*M* *_r_* = 348.30
Triclinic, 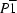

*a* = 6.980 (1) Å
*b* = 7.615 (1) Å
*c* = 8.294 (1) Å
α = 106.376 (3)°
β = 93.698 (3)°
γ = 109.085 (3)°
*V* = 393.7 (1) Å^3^
*Z* = 1
Mo *K*α radiation
μ = 0.11 mm^−1^
*T* = 293 K
0.42 × 0.37 × 0.32 mm

#### Data collection

Bruker SMART CCD area-detector diffractometer
Absorption correction: multi-scan (*SADABS*; Sheldrick, 1996 ▶) *T*_min_ = 0.782, *T*_max_ = 1.000
2165 measured reflections
1529 independent reflections
1259 reflections with *I* > 2σ(*I*)
*R*_int_ = 0.065

#### Refinement

*R*[*F* ^2^ > 2σ(*F* ^2^)] = 0.047
*wR*(*F* ^2^) = 0.137
*S* = 1.07
1529 reflections
119 parameters
H-atom parameters constrained
Δρ_max_ = 0.19 e Å^−3^
Δρ_min_ = −0.21 e Å^−3^

### 

Data collection: *SMART* (Bruker, 2001 ▶); cell refinement: *SAINT* (Bruker, 2000 ▶); data reduction: *SHELXTL* (Sheldrick, 2008 ▶); program(s) used to solve structure: *SHELXS97* (Sheldrick, 2008 ▶); program(s) used to refine structure: *SHELXL97* (Sheldrick, 2008 ▶); molecular graphics: *SHELXTL* (Sheldrick, 2008 ▶); software used to prepare material for publication: *SHELXTL* (Sheldrick, 2008 ▶).

## Supplementary Material

Crystal structure: contains datablocks global, I. DOI: 10.1107/S1600536809035247/im2138sup1.cif

Structure factors: contains datablocks I. DOI: 10.1107/S1600536809035247/im2138Isup2.hkl

Additional supplementary materials:  crystallographic information; 3D view; checkCIF report

## Figures and Tables

**Table 1:** Hydrogen-bond geometry (Å, °)

*D*—H⋯*A*	*D*—H	H⋯*A*	*D*⋯*A*	*D*—H⋯*A*
C9—H9⋯N1^i^	0.93	2.50	3.410 (2)	166

Symmetry code: (i) 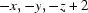.

**Fig. 1.**
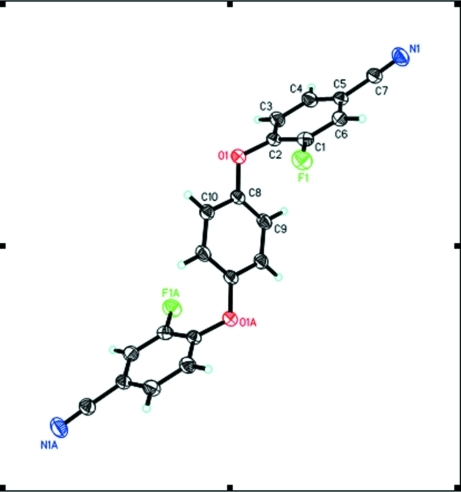
Molecular structure of title compound with the atomic labeling scheme. Displacement ellipsoids are drawn at the 50% probability level.

**Fig. 2.**
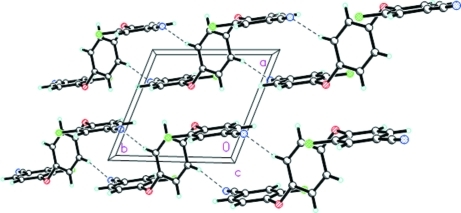
A partial packing diagram of title compound. Hydrogen bonds are shown as dashed lines. [Symmetry code: (i) -x, -y, -z + 2].

## supplementary crystallographic information

### Comment

There has been growing interest in the study of hydroquinone derivatives which are important intermediates in the synthesis of herbicides (Liu, 2002. Bao *et al.*, 2007). Only a few compounds of this kind have been structurally characterized so far. As part of our studies, we have synthesized the title compound from hydroquinone and 3,4-difluorobenzonitrile and report it's crystal structure in this article.

The crystal structure of the title compound (Fig. 1) utilizes the symmetry of the crystallographic inversion center similarily to a related selenium compound (Sørensen *et al.*, 2009). The two terminal (C1—C7) phenyl ring and the central ring together with the attached oxygen (C8—C10/O1) form three planes. Due to crystallograhic symmetry the two terminal phenyl rings are coplanar. The terminal (C1—C7) phenyl ring plane and the central ring plane enclose a dihedral angle of 77.8 (3)°. Otherwise, the molecule is bent with the C2—O1—C8 angle of 118.25°.

In the crystal structure, intermolecular C—H···N hydrogen bonds (Tab.1) connect neighboring molecules with each other to form a one-dimensional chain that stretches along the *c* axis (Fig.2).

### Experimental

A DMF (10 ml) solution of hydroquinone (1 mmol) and 3,4-difluorobenzonitrile (2 mmol) was heated to 70°C in the presence of KOH and stirred for 37 h. Then the mixture was washed with water (30 ml) and extracted with ethyl acetate (three times). The organic solvent was removed under reduced pressure. Afterwards the product was purified by column chromatography on silica (pentane - ethyl acetate mixtures). Single crystals were obtained by slow evaporation of the solvent of an ethanolic solution at room temperature.

### Refinement

H atoms were placed in calculated positions with C—H=0.93 Å. All H atoms were included in the final cycles of refinement using a riding model, with *U*_iso_(H)=1.2*U*_eq_ of the carrier atoms.

### Crystal data

**Table:**

C_20_H_10_F_2_N_2_O_2_	*Z* = 1
*M**_r_* = 348.30	*F*(000) = 178
Triclinic, *P*1	*D*_x_ = 1.469 Mg m^−^^3^
Hall symbol: -P 1	Mo *K*α radiation, λ = 0.71073 Å
*a* = 6.980 (1) Å	Cell parameters from 1140 reflections
*b* = 7.615 (1) Å	θ = 5.2–54.9°
*c* = 8.294 (1) Å	µ = 0.11 mm^−^^1^
α = 106.376 (3)°	*T* = 293 K
β = 93.698 (3)°	Prismatic, colorless
γ = 109.085 (3)°	0.42 × 0.37 × 0.32 mm
*V* = 393.7 (1) Å^3^	

### Data collection

**Table:**

Bruker SMART CCD area-detector diffractometer	1529 independent reflections
Radiation source: fine-focus sealed tube	1259 reflections with *I* > 2σ(*I*)
graphite	*R*_int_ = 0.065
φ and ω scans	θ_max_ = 26.0°, θ_min_ = 2.6°
Absorption correction: multi-scan (*SADABS*; Sheldrick, 1996)	*h* = −8→7
*T*_min_ = 0.782, *T*_max_ = 1.000	*k* = −9→8
2165 measured reflections	*l* = −10→10

### Refinement

**Table:**

Refinement on *F*^2^	Secondary atom site location: difference Fourier map
Least-squares matrix: full	Hydrogen site location: inferred from neighbouring sites
*R*[*F*^2^ > 2σ(*F*^2^)] = 0.047	H-atom parameters constrained
*wR*(*F*^2^) = 0.137	*w* = 1/[σ^2^(*F*_o_^2^) + (0.0836*P*)^2^] where *P* = (*F*_o_^2^ + 2*F*_c_^2^)/3
*S* = 1.07	(Δ/σ)_max_ = 0.001
1529 reflections	Δρ_max_ = 0.19 e Å^−^^3^
119 parameters	Δρ_min_ = −0.21 e Å^−^^3^
0 restraints	Extinction correction: *SHELXL97* (Sheldrick, 2008), Fc^*^=kFc[1+0.001xFc^2^λ^3^/sin(2θ)]^-1/4^
Primary atom site location: structure-invariant direct methods	Extinction coefficient: 0.027 (6)

### Special details

**Table:**

Geometry. All e.s.d.'s (except the e.s.d. in the dihedral angle between two l.s. planes) are estimated using the full covariance matrix. The cell e.s.d.'s are taken into account individually in the estimation of e.s.d.'s in distances, angles and torsion angles; correlations between e.s.d.'s in cell parameters are only used when they are defined by crystal symmetry. An approximate (isotropic) treatment of cell e.s.d.'s is used for estimating e.s.d.'s involving l.s. planes.
Refinement. Refinement of *F*^2^ against ALL reflections. The weighted *R*-factor *wR* and goodness of fit *S* are based on *F*^2^, conventional *R*-factors *R* are based on *F*, with *F* set to zero for negative *F*^2^. The threshold expression of *F*^2^ > σ(*F*^2^) is used only for calculating *R*-factors(gt) *etc*. and is not relevant to the choice of reflections for refinement. *R*-factors based on *F*^2^ are statistically about twice as large as those based on *F*, and *R*- factors based on ALL data will be even larger.

### Fractional atomic coordinates and isotropic or equivalent isotropic displacement parameters (Å^2^)

**Table:**

	*x*	*y*	*z*	*U*_iso_*/*U*_eq_	
O1	0.31400 (15)	0.41880 (17)	0.64633 (14)	0.0546 (4)	
N1	0.2645 (2)	0.0341 (2)	1.2835 (2)	0.0706 (5)	
F1	0.21076 (16)	0.59370 (13)	0.94706 (13)	0.0663 (4)	
C1	0.2380 (2)	0.4219 (2)	0.9251 (2)	0.0463 (4)	
C2	0.2823 (2)	0.3307 (2)	0.77070 (18)	0.0443 (4)	
C3	0.3137 (2)	0.1573 (2)	0.7484 (2)	0.0520 (4)	
H3	0.3429	0.0941	0.6448	0.062*	
C4	0.3023 (2)	0.0761 (2)	0.8780 (2)	0.0524 (4)	
H4	0.3229	−0.0419	0.8619	0.063*	
C5	0.2601 (2)	0.1714 (2)	1.03224 (18)	0.0460 (4)	
C6	0.2256 (2)	0.3453 (2)	1.05636 (19)	0.0483 (4)	
H6	0.1949	0.4084	1.1592	0.058*	
C7	0.2590 (2)	0.0934 (2)	1.1718 (2)	0.0535 (4)	
C8	0.1518 (2)	0.4580 (2)	0.57622 (16)	0.0424 (4)	
C9	−0.0514 (2)	0.3477 (2)	0.56761 (18)	0.0478 (4)	
H9	−0.0856	0.2450	0.6129	0.057*	
C10	0.2042 (2)	0.6087 (2)	0.50919 (18)	0.0472 (4)	
H10	0.3420	0.6816	0.5152	0.057*	

### Atomic displacement parameters (Å^2^)

**Table:**

	*U*^11^	*U*^22^	*U*^33^	*U*^12^	*U*^13^	*U*^23^
O1	0.0475 (6)	0.0766 (8)	0.0542 (7)	0.0254 (5)	0.0116 (5)	0.0384 (6)
N1	0.0775 (11)	0.0788 (11)	0.0601 (9)	0.0206 (8)	0.0085 (8)	0.0390 (8)
F1	0.0825 (8)	0.0573 (7)	0.0671 (7)	0.0349 (5)	0.0118 (5)	0.0209 (5)
C1	0.0428 (8)	0.0463 (8)	0.0503 (9)	0.0176 (6)	0.0023 (6)	0.0158 (7)
C2	0.0385 (7)	0.0561 (9)	0.0407 (8)	0.0150 (6)	0.0028 (6)	0.0222 (7)
C3	0.0585 (9)	0.0626 (10)	0.0412 (8)	0.0290 (8)	0.0095 (7)	0.0172 (7)
C4	0.0578 (9)	0.0548 (9)	0.0510 (9)	0.0258 (7)	0.0070 (7)	0.0206 (7)
C5	0.0397 (8)	0.0560 (9)	0.0429 (8)	0.0144 (6)	0.0018 (6)	0.0211 (7)
C6	0.0451 (8)	0.0586 (9)	0.0389 (8)	0.0177 (7)	0.0049 (6)	0.0141 (7)
C7	0.0481 (9)	0.0621 (10)	0.0496 (9)	0.0149 (7)	0.0036 (7)	0.0238 (8)
C8	0.0453 (8)	0.0489 (8)	0.0338 (7)	0.0167 (6)	0.0029 (6)	0.0160 (6)
C9	0.0496 (9)	0.0461 (8)	0.0472 (9)	0.0093 (6)	0.0032 (6)	0.0247 (7)
C10	0.0401 (8)	0.0512 (9)	0.0454 (8)	0.0058 (6)	0.0033 (6)	0.0217 (7)

### Geometric parameters (Å, °)

**Table:**

O1—C2	1.3731 (16)	C4—H4	0.9300
O1—C8	1.3943 (16)	C5—C6	1.385 (2)
N1—C7	1.143 (2)	C5—C7	1.442 (2)
F1—C1	1.3469 (17)	C6—H6	0.9300
C1—C6	1.368 (2)	C8—C10	1.3678 (19)
C1—C2	1.381 (2)	C8—C9	1.377 (2)
C2—C3	1.372 (2)	C9—C10^i^	1.385 (2)
C3—C4	1.378 (2)	C9—H9	0.9300
C3—H3	0.9300	C10—C9^i^	1.385 (2)
C4—C5	1.384 (2)	C10—H10	0.9300
			
C2—O1—C8	118.23 (11)	C6—C5—C7	119.29 (14)
F1—C1—C6	119.41 (14)	C1—C6—C5	118.30 (14)
F1—C1—C2	118.49 (13)	C1—C6—H6	120.8
C6—C1—C2	122.08 (14)	C5—C6—H6	120.8
C3—C2—O1	119.59 (13)	N1—C7—C5	177.94 (17)
C3—C2—C1	118.81 (13)	C10—C8—C9	120.96 (13)
O1—C2—C1	121.32 (13)	C10—C8—O1	116.41 (12)
C2—C3—C4	120.60 (14)	C9—C8—O1	122.58 (12)
C2—C3—H3	119.7	C8—C9—C10^i^	119.29 (13)
C4—C3—H3	119.7	C8—C9—H9	120.4
C3—C4—C5	119.53 (15)	C10^i^—C9—H9	120.4
C3—C4—H4	120.2	C8—C10—C9^i^	119.75 (13)
C5—C4—H4	120.2	C8—C10—H10	120.1
C4—C5—C6	120.67 (13)	C9^i^—C10—H10	120.1
C4—C5—C7	120.00 (15)		
			
C8—O1—C2—C3	123.31 (15)	C2—C1—C6—C5	0.4 (2)
C8—O1—C2—C1	−62.84 (18)	C4—C5—C6—C1	−1.1 (2)
F1—C1—C2—C3	178.88 (13)	C7—C5—C6—C1	176.65 (13)
C6—C1—C2—C3	0.4 (2)	C4—C5—C7—N1	80 (5)
F1—C1—C2—O1	5.0 (2)	C6—C5—C7—N1	−98 (5)
C6—C1—C2—O1	−173.52 (13)	C2—O1—C8—C10	154.42 (13)
O1—C2—C3—C4	173.66 (13)	C2—O1—C8—C9	−28.1 (2)
C1—C2—C3—C4	−0.3 (2)	C10—C8—C9—C10^i^	−0.4 (2)
C2—C3—C4—C5	−0.4 (2)	O1—C8—C9—C10^i^	−177.78 (13)
C3—C4—C5—C6	1.2 (2)	C9—C8—C10—C9^i^	0.4 (2)
C3—C4—C5—C7	−176.60 (14)	O1—C8—C10—C9^i^	177.93 (12)
F1—C1—C6—C5	−178.13 (13)		

Symmetry codes: (i) −*x*, −*y*+1, −*z*+1.

### Hydrogen-bond geometry (Å, °)

**Table:**

*D*—H···*A*	*D*—H	H···*A*	*D*···*A*	*D*—H···*A*
C9—H9···N1^ii^	0.93	2.50	3.410 (2)	166

Symmetry codes: (ii) −*x*, −*y*, −*z*+2.
